# Abuses of Amalgams and Their Treatment

**Published:** 1907-06-15

**Authors:** N. K. Garhart


### Abuses of Amalgams and Their Treatment.

BY N. K. GARHART.

Amalgam fillings fail for several reasons. First, because most dentists do not charge a fee for them that will justify the use of good amalgam and the necessary care and skill to properly insert them.

Secondly, most amalgams used are cheaply and indifferently manufactured, and good results can not be expected from them.

Thirdly, most of the men who buy and use cheap amalgam are cheap manipulators, and they could not be expected to get good results even if they could be induced to use good amalgams.

Fourthly, Even good dentists have not yet learned that it is necessary when making approximal fillings, to insert a matrix in order that the amalgam be properly placed in the cavity.

Fifthly. We learn so slowly, that after all these years many of us believe that we can insert amalgam in the teeth without securing absolute dryness of the cavity. The rubber dam is essential.

Sixthly. The alloy must be more pertectly amalgamated than we are accustomed to make it. Thorough amalgamation and expression of all excess mercury is necessary.

Seventhly. The cavity must be prepared in such a manner that the amalgam can be placed definitely and quickly, and there should be no attenuated margins to be fractured under the stress of mastication.

Eighthly. The amalgam should be packed when it is of the proper consistence, neither when it is too soft nor atter it has begun to set.

Ninthly. The instrument used should fit the case and be adapted for strong torce. The faces should be as large as are admissable, and be applied in the proper manner to force the material to place with a few positive and well directed motions.

Tenthly. Amalgam is not indicated for all kinds of cavities, and should not be depended upon as a universal filling material

The public has been educated to believe that amalgam is a cheap and poor filling material. This must be changed, and can best be done by skillful and conscientious operators, who will make themselves intelligent on the material and use it properly and in the right places.

Amalgam made to contain from 60 to 68 percent of silver can be depended upon for accurate results if made by a skilltul and conscientious manufacturer, and such manutacturers we have, who should be encouraged by the profession, even though their product may exceed in price that of the men who hire their alloys made by any one who will do it the cheapest.

The technique of manipulation by the dentist is ot the greatest importance. The best alloys are the so-called hard alloys, and they are the most difficult to manipulate. The mortar is the best way I think to secure good amalgamation. Use an excess of mercury to secure a perfect mix, but express all that is possible before beginning to pack into the cavity. I prefer to take the mix out of the mortar and place it in a piece of chamois skin and express all the mercury I can, and then return to the mortar and triturate and again express the free mercury, and it is ready to pack. I sometimes mix and retriturate and express the mercury three or four times, and sometimes make two or three mixes for one filling.

Never leave a soft mass of amalgam on the surface of the filling; scrape it out and pack in harder amalgam. Leave the fillings over full and when the amalgam has set dress it to the proper contour. Shrinkage, spheroiding and change of form are the three greatest amalgam faults; all of these are due to faulty manipulation of the amalgam in mixing. The high-grade alloys do not amalgamate easily, hence care should be used to produce perfect mixtures. I get the best results by using enough mercury and expressing all excess carefully and thoroughly.

### DISCUSSION.

Dr. H. T. Smith. Our amalgams used to contain a large quantity of tin, and many of them today do; this makes them set slowly and they are very easy for the dentist to manipulate, hence they are still popular and sell well. But all such fillings are liable to give unsatisfactory results as well as to lead to slovenly methods of manipulation. If an amalgam shrinks much during its primary setting, as the soft amalgams are likely to do, there is so much loss of material that the secondary expansion never compensates it, hence loose, leaky fillings and recurrent decay. It may be desirable to have the proper weights of mercury and alloy fixed and always adhered to, but even this will not secure the best results if the dentist fails to mix properly or manipulate the amalgam when introducing it into the tooth.

Dr. J. R. Callahan called attention to the fact that it was not possible to insert and properly polish amalgam fillings at the same sitting. At least twenty-four hours was necessary to get sufficient hardness to make a high polish possible. He suggested that it was a good plan to place the amalgam on a slab after mixing it in the mortar and squeezing out the excess mercury, and spatulate it vigorously with a strong cement spatula. In packing amalgam, he explained his method of packing with the Herbst rotary burnishers in the engine. He believes he gets better wall contacts and more homogeneous filling than can be made with the hand pressure instruments.

Dr. N. S. Hoff. The essayist implies that amalgams are now to be had that are perfectly made from the manufacturer's standpoint, and that it is the dentist who is now at fault. We have been for years trying to improve our technique so that we may work amalgam so that it will save the teeth, and we are still far from being able to say that amalgam is the ideal filling material. From my own experience as an operator, and my observations of the efforts of manufacturers to supply a proper material, I am convinced that the dentists are not wholly to blame for the failures of amalgam as a filling material. I am not at all sure that our chemists have provided us with a material that is workable and safe to depend upon. As the essayist well says there are a number of good alloys on the market, but the general practitioner does not know which are the good ones and which should be avoided. It is essential that our alloys should be made after a good and true formula, but it is equally essential that the formula should be accurately and conscientiously compounded, and that every step in the process should be scientifically taken and the resultant alloy be properly tested out before it is marketed. Too many of our alloys are made by the office boy or the hired man. If our best operators find after the most careful and pains-taking efforts that the best alloys they can buy fail to bring satisfactory results can any one be blamed but the manufacturers for faults that are purely of the material?

